# Comparison of short-term efficacy between robot-assisted and uniportal video-assisted thoracoscopic surgery for right upper lobectomy in non-small cell lung cancer

**DOI:** 10.3389/fsurg.2026.1773128

**Published:** 2026-04-13

**Authors:** Xiangping Zhu, Ke Xu, Xiaoyan Feng, Rongsheng Xiong

**Affiliations:** Department of Thoracic Surgery, Nanxishan Hospital of Guangxi Zhuang Autonomous Region (the Second People's Hospital of Guangxi Zhuang Autonomous Region), Guilin, Guangxi, China

**Keywords:** non-small cell lung cancer, NSCLC, right upper lobectomy, robot, uniportal video-assisted thoracoscopic surgery

## Abstract

**Objective:**

To compare the short-term efficacy of robot-assisted and uniportal video-assisted thoracoscopic surgery (U-VATS) for right upper lobectomy in treating non-small cell lung cancer (NSCLC).

**Methods:**

99 early-stage NSCLC patients from Nanxishan Hospital of Guangxi Zhuang Autonomous Region who underwent surgery between July 2022 and December 2024, were selected and grouped based on the surgical approach: patients undergoing da Vinci robot-assisted right upper lobectomy (da Vinci group) and patients undergoing U-VATS right upper lobectomy (U-VATS group). Clinical data were compared between the two groups, including baseline data, efficacy, surgical indicators, postoperative complications, and survival curves.

**Results:**

There were no statistically significant differences in baseline data between the two groups (*P* > 0.05). The efficacy between the two groups showed no statistical significance (*P* > 0.05); however, the R0 was higher in the da Vinci robot group at 86.00% compared to 73.47% in the U-VATS group. There were no statistically significant differences in terms of surgical time, intraoperative blood loss, chest tube drainage, duration of drainage tube placement, and length of postoperative hospital stay between the two groups (*P* > 0.05). The da Vinci robot group had a higher number of lymph node dissections than the U-VATS group (*P* < 0.05). The incidence of postoperative complications showed no statistically significant difference between the two groups (*P* > 0.05). There were no cases of loss to follow-up among the 99 patients. The survival rate was 89.80% in the U-VATS group and 96.00% in the da Vinci robot group, with no statistical significance (*P* > 0.05).

**Conclusion:**

Compared to U-VATS, da Vinci robot-assisted right upper lobectomy for early-stage NSCLC patients demonstrates similar safety and operability, with a significantly higher number of lymph node dissections. There were no significant differences in surgical time, intraoperative blood loss, postoperative chest tube drainage, duration of drainage tube placement, length of postoperative hospital stay, and incidence of postoperative lung infections between the two approaches.

## 1 Introduction

Globally, lung cancer has the highest incidence and mortality rate among male malignant tumors and ranks second among females (1). In China, the incidence and mortality rates of lung cancer also rank highest among malignant tumors, with only 30% of early and locally advanced lung cancer cases having the opportunity for curative surgical treatment (2). Non-small cell lung cancer (NSCLC) accounts for the majority of lung cancer cases and is primarily treated by surgical resection, often in combination with chemotherapy, radiotherapy, targeted therapy, and immunotherapy. Surgical approaches have evolved from open surgeries to thoracoscopic minimally invasive surgeries, and more recently to robot-assisted surgeries. Since the first da Vinci robot-assisted pulmonary lobectomy performed by Melfi et al. (3), the volume of such surgeries in mainland China has surged, starting with the first case completed by Director Luo Qingquan in 2009. Following the introduction of the Da Vinci S system in 2006, there has been an increase in reports of robot-assisted thoracic surgeries (4–6). In 2009, Huang Jia et al. reported the first case of robot-assisted pulmonary lobectomy in mainland China, and Zhao et al. (7) also reported on five cases of robot-assisted pulmonary lobectomies. A meta-analysis by Liang et al. (8) involving 7,438 patients further confirmed the safety and efficacy of robot-assisted surgery in the curative treatment of lung cancer, with lower conversion rates and perioperative mortality rates compared to traditional thoracoscopic surgeries. The da Vinci robot system operates by inputting three-dimensional coordinates and other numerical data, which are then converted and executed by the robot, demanding high computational response times. In the future, with the improvement of internet fiber optic transmission speeds, remote robotic surgeries are expected to become a reality. Additionally, current multi-port robotic operation modes may evolve into single-port operations, which are more patient-friendly, thus propelling the rapid development of new technologies. The application of robotic systems is pushing minimally invasive techniques to new heights.

### 1.1 Research objects

Early-stage NSCLC patients from Nanxishan Hospital of Guangxi Zhuang Autonomous Region between July 2022 and December 2024 were selected and grouped based on the surgical approach. The groups included early-stage NSCLC patients undergoing da Vinci robot-assisted right upper lobectomy (da Vinci group) and those undergoing uniportal video-assisted thoracoscopic surgery (U-VATS group). Patients in the U-VATS group (*n* = 49) and patients in the da Vinci group (*n* = 50) were included after obtaining voluntary participation and informed consent following approval from the hospital's ethics committee.

Inclusion Criteria: (1) Patients undergoing da Vinci robot-assisted and U-VATS right upper lobectomy with systematic lymph node dissection during the same period. (2) Preoperative routine tests and examinations showing no contraindications for surgery, and no distant metastases. (3) No prior neoadjuvant therapy. (4) No history of chest trauma or chest surgery. (5) Absence of a history of other tumors. (6) Age between 18 and 75 years; maximum voluntary ventilation (MVV) ≥ 40% of predicted value. (7) All patients received general anesthesia with combined inhalation and intravenous anesthesia, double-lumen endotracheal intubation, and ventilation of the healthy lung. (8) Postoperative pathology diagnosis of NSCLC; Tumor staging according to the 8th edition TNM staging criteria of the Union for International Cancer Control/American Joint Committee on Cancer (UICC/AJCC).

Exclusion Criteria: (1) Age <18 years or >75 years. (2) Surgical procedures involving bronchial or pulmonary artery formation. (3) Pathological diagnosis of benign tumors or small cell lung cancer. (4) Conversion to open surgery or extensive dense adhesions of the pleura ≥ 50%. (5) Patients who refused relevant treatment for various reasons or had incomplete clinical data records.

### 1.2 Methods

#### 1.2.1 U-VATS group

The patients were positioned in a left lateral decubitus position with the chest elevated. A single incision of 3∼4 cm was made at the 4th or 5th intercostal space along the right anterior axillary line for the insertion of a disposable port fixation device, serving as both the thoracoscope observation port and working port.

#### 1.2.2 Da vinci robot group

The patients were positioned in a left lateral decubitus position with the right arm raised and bent at the elbows in front of the head, and the lower chest was elevated with a folded towel. The “8-8-5-7” port design method was used: a 12 mm trocar was inserted at the 8th intercostal space along the posterior axillary line for the camera port; 8 mm trocars were placed at the 8th intercostal space along the midaxillary line and the 5th intercostal space along the anterior axillary line for instrument ports connected to the robotic arms; a 3-4 cm incision was made at the 7th intercostal space along the midaxillary line and a disposable port fixation device was inserted as an auxiliary operating port. Lymph Node Dissection: If preoperative pathology or intraoperative frozen section pathology indicated malignancy in both groups, a lung lobectomy with systematic lymph node dissection was performed directly. The lymph node dissection range included nodes 2nd ∼4th groups and the 7th ∼12th groups. Closure and Chest Tube Drainage Placement: Sterile injection water was poured into the chest cavity, and the anesthesiologist was instructed to fully inflate the lungs while ensuring no air leakage from the bronchial stump or lung tissue and no significant bleeding or oozing in the surgical field. In the da Vinci robot group, 22# silicone chest tubes were placed at the 5th intercostal space along the anterior axillary line and the 8th intercostal space along the posterior axillary line. In the U-VATS group, 22# silicone chest tubes were placed through the thoracoscopic incision.

### 1.3 Observational indicators

(1) Comparison of baseline data between the two groups. (2) Based on the comparison of short-term efficacy based on surgical status, it is divided into complete tumor resection (R0): no cancer cells can be found at the cutting edge under the microscope, and there is no residual cancer cell under the naked eye or microscope. The lesion is completely removed. Microscopic residue (R1): All large lesions were removed, and there were cancer cells at the cutting edge under the microscope. Tumor residue (R2): Visible margin tissue with the naked eye.(3) Comparison of surgical metrics between the two groups, including operation time, intraoperative blood loss, number of dissected lymph nodes, and chest tube drainage volume (on the second day postoperatively). (4) Comparison of postoperative complication rates among patients, including postoperative air leak, inflammation, and pleural effusion. Postoperative Air Leak: After chest surgery, air leaks into the chest cavity from damaged lung tissue or pleural cavity, causing air accumulation. This is often due to surgical procedures that open the airway on the lung surface or bronchial stump, affecting lung expansion and respiratory function. Inflammation: During chest surgery, tissue damage activates the immune system and triggers a defense response, resulting in local redness, swelling, pain, and functional impairment. The whole body may also experience fever, elevated white blood cells, and severe SIRS, affecting multi organ function. Pleural Infusion: Excessive accumulation of fluid in the chest cavity after surgery can occur for various reasons, including compression of lung tissue and difficulty breathing. Postoperative pulmonary infection: postoperative pulmonary infectious diseases, most patients have fever and other symptoms, and severe respiratory failure endangers life. (5) Comparison of survival curves and progression-free survival between the two groups.

### 1.4 Statistical analysis

The collected experimental data was analyzed using SPSS 27.0 (International Business Machines Corporation, Armonk, New York, USA). The Shapiro–Wilk test was used for normality testing. Normally distributed continuous data was presented as `X ± S and compared using independent sample t-tests. Non-normally distributed data was expressed as MQ2 (Q1, Q3), and analyzed using the Mann–Whitney U test. Categorical data was presented as counts or rates and compared using *χ*2 test or Fisher's exact test, with statistical significance set at *P* < 0.05.

## 2 Results

### 2.1 Comparison of baseline data between Two groups

Comparison of baseline data between the two groups, including age, gender, height, weight, BMI, pathological type, differentiation degree, TNM staging, and lymph node metastasis, showed no statistically significant differences (*P* > 0.05), as shown in Table 1.

**Table 1:** Comparison of baseline data between two groups.

Indicator	Classification	U-VATS Group (*n* = 49)	Da Vinci Robot Group (*n* = 50)	*t/χ*^2^ Value	*P* Value
Age (years)		63.59 ± 7.62	61.10 ± 7.22	1.669	0.098
Gender	Male	26 (53.06)	33 (66.00)	1.721	0.190
	Female	23 (46.94)	17 (34.00)		
Height (cm)		161.80 ± 7.88	161.92 ± 7.51	0.078	0.938
Weight (kg)		61.61 ± 11.56	61.56 ± 10.61	0.022	0.982
BMI (kg/m2)		23.47 ± 3.51	23.39 ± 3.04	0.121	0.904
Pathological Type	Adenocarcinoma	44 (89.80)	38 (76.00)	6.520	0.089
	Squamous cell carcinoma	5 (10.20)	6 (12.00)		
	Adenosquamous carcinoma	0 (0.00)	1 (2.00)		
	Others	0 (0.00)	6 (10.00)		
Differentiation Degree	Poorly Differentiated	11 (22.45)	10 (20.00)	1.751	0.626
	Moderately Differentiated	21 (42.86)	25 (50.00)		
	Highly Differentiated	9 (18.37)	5 (10.00)		
	Others	8 (16.33)	10 (20.00)		
TNM Staging	Stage I	36 (73.47)	39 (78.00)	0.312	0.856
	Stage II	4 (8.16)	3 (6.00)		
	Stage III	9 (18.37)	8 (16.00)		
Lymph Node Metastasis	Yes	7 (14.29)	9 (18.00)	0.252	0.616
	No	42 (85.71)	41 (82.00)		

### 2.2 Comparison of efficacy between Two groups

The comparison of efficacy between the two groups showed no statistical significance (*P* > 0.05). However, based on the data, the R0 in the da Vinci robot group was 86.00%, slightly higher than the U-VATS group at 73.47%, as shown in Table 2.

**Table 2:** Comparison of efficacy between two groups.

Grouping	Number of Cases	R0	R1	R2
U-VATS Group	49	36 (73.47)	12 (24.49)	1 (2.04)
Da Vinci Robot Group	50	43 (86.00)	7 (14.00)	0 (0.00)
*χ*^2^ Value		2.410		
*P* Value		0.121		

### 2.3 Comparison of surgical indicators between Two groups

The comparison of surgical indicators between the two groups, including surgical time, intraoperative blood loss, chest tube drainage volume, duration of drainage tube placement, and length of postoperative hospital stay, showed no statistically significant differences (*P* > 0.05). However, the number of lymph nodes dissected in the da Vinci robot group was higher than in the U-VATS group (*P* < 0.05), as shown in Table 3.

**Table 3:** Comparison of surgical indicators between two groups.

Indicator	U-VATS Group (*n* = 49)	Da Vinci Robot Group (*n* = 50)	*t/Z*	*P*
Surgical Time (min)	173.86 ± 48.33	192.76 ± 57.08	1.776	0.079
Intraoperative Blood Loss (mL)	200.00 (100.00, 300.00)	100.00 (100.00, 200.00)	−1.526	0.127
Number of Lymph Nodes Dissected	11.00 (7.00, 15.50)	12.50 (10.00, 18.00)	−2.241	0.025
Chest Tube Drainage Volume (mL)	260.00 (195.00, 420.00)	255.00 (200.00, 400.00)	−0.372	0.710
Duration of Drainage Tube Placement (days)	4.00 (3.00, 6.00)	3.00 (2.00, 4.25)	−1.818	0.069
Length of Postoperative Hospital Stay (days)	8.00 (6.00, 10.00)	7.00 (7.00, 9.00)	−0.475	0.634

### 2.4 Comparison of postoperative complication rates between Two groups

The comparison of postoperative complication rates between the two groups showed no statistically significant differences (*P* > 0.05), as illustrated in Table 4.

**Table 4:** Comparison of postoperative complication rates between two groups.

Grouping	Number of Cases	Postoperative Air Leak	Inflammation	Pleural Effusion	Postoperative Pulmonary Infection
U-VATS Group	49	1（2.04）	1（2.04）	1（2.04）	4（8.16）
Da Vinci Robot Group	50	0（0.00）	1（2.00）	0（0.00）	1（2.00）
*χ*^2^ Value		-			
*P* Value		0.075			

Fisher's exact test is used for chi square analysis here.

### 2.5 Survival curve analysis of One-year mortality rates between Two groups

**Figure 1:**
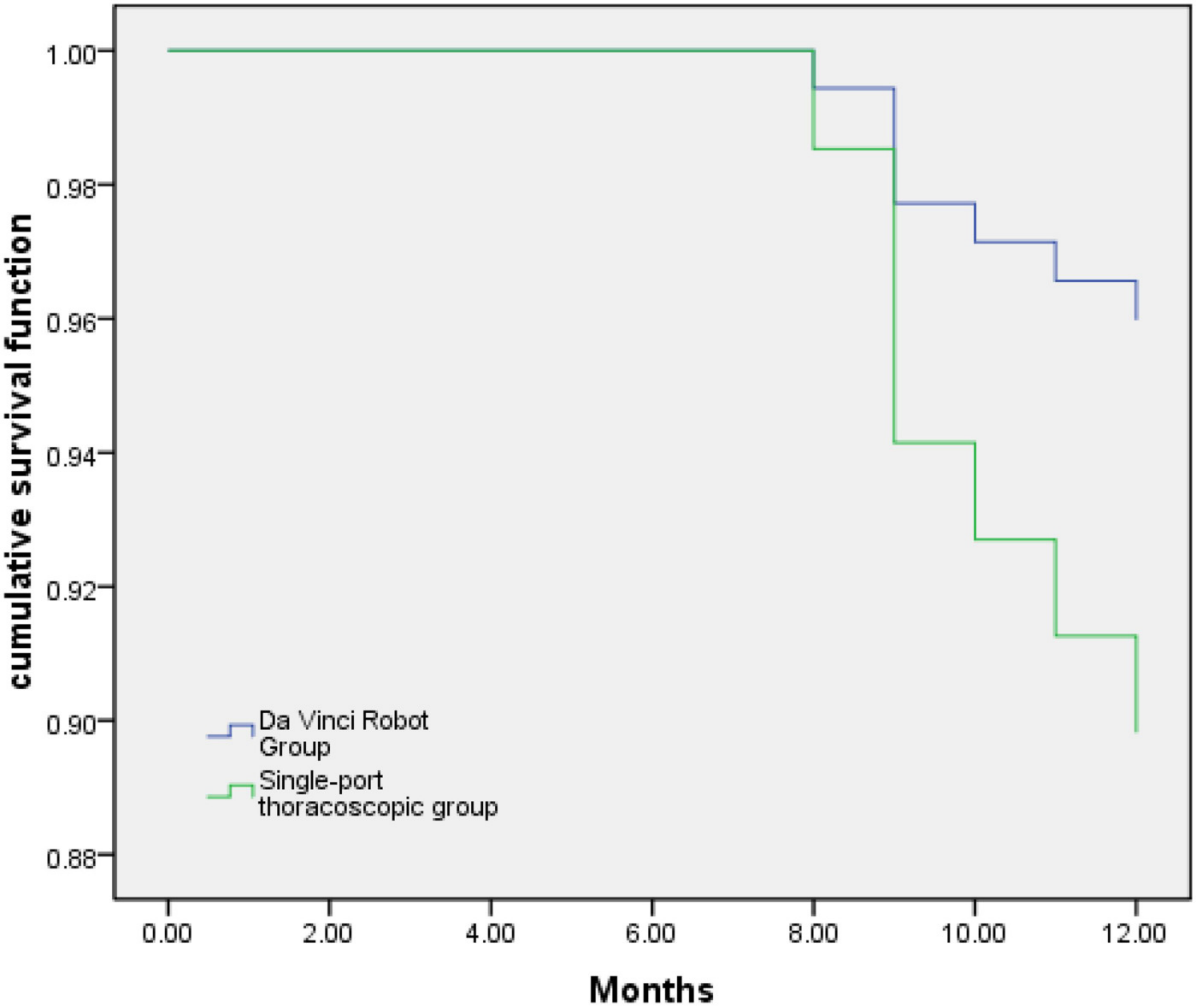
Survival curve analysis of one-year mortality rates between two groups. Log Rank (Mantel-Cox): *χ*^2^ Value = 1.466, *P* Value = 0.233.

All 99 patients had no cases of loss to follow-up. The one-year survival rate was 89.80% in the U-VATS group and 96.00% in the da Vinci robot group, with no statistically significant difference (*P* > 0.05),This survival analysis is an exploratory study with a 1-year period, which represents short-term (1-year) survival. The number of events (deaths) may be low, resulting in insufficient ability for survival analysis. Further follow-up is needed for comparison in the future. as depicted in Figure 1.

## 3 Discussion

In the field of lung cancer treatment, surgical intervention for NSCLC has always been a focal point of research. With continuous advancements in medical technology, surgical approaches have evolved from traditional open surgeries to minimally invasive procedures primarily supported by thoracoscopic assistance, and now to the rapidly emerging field of robot-assisted surgeries. Particularly, the comparative efficacy of robot-assisted right upper lobectomy and U-VATS right upper lobectomy in treating NSCLC has become a hot topic of interest in the field of thoracic surgery (9).

From the perspective of surgical safety, both robot-assisted and U-VATS right upper lobectomy procedures have demonstrated a high level of proficiency in this study. We selected 99 early-stage NSCLC patients from July 2022 to December 2024, dividing them into the da Vinci robot-assisted group (da Vinci group) and the uniportal video-assisted thoracoscopic surgery group (U-VATS group). The research results indicated that there was no statistically significant difference in the occurrence of postoperative complications between the two groups. This suggests that both robot-assisted and U-VATS surgeries can effectively control surgical risks in the short term, ensuring patient safety. This is attributed to both procedures falling within the realm of minimally invasive surgery. Compared to traditional open surgeries, these approaches result in less physical trauma to the patients, reducing physiological stress responses after surgery and lowering the likelihood of postoperative complications (10). For instance, traditional open chest surgeries involve cutting through multiple muscle groups in the chest and back, and spreading the ribs, causing significant trauma leading to severe postoperative pain and increased complications. In contrast, robot-assisted and U-VATS surgeries involve small incisions, avoiding these issues and laying a solid foundation for patients' postoperative recovery (11, 12).

In terms of surgical feasibility, both surgical approaches have their unique characteristics. Robot-assisted surgery offers distinct advantages with its robotic arms not operating solely through manual mechanical force transmission but rather with the input of various numerical values such as specific three-dimensional coordinates by the surgeon into the system. Through digital calculations and conversions, the robot performs the surgical procedure. This precision and stability in surgical operations are enhanced, particularly when dealing with complex anatomical structures, where the flexibility and controllability of the robotic arms prove crucial (13). For instance, during lymph node dissection, robot-assisted surgery can precisely remove lymph nodes, reducing damage to surrounding tissues. In this study, the da Vinci robot group had a higher number of lymph nodes dissected compared to the U-VATS group, highlighting the advantage of robot-assisted surgery in terms of operability. Previous studies by Pan et al. (14) have also shown that the number of lymph nodes dissected in robot-assisted surgeries is higher than in thoracoscopic-assisted surgeries, further validating the results of this study. On the other hand, U-VATS surgery, despite having a relatively narrow operating space and requiring a high level of technical proficiency from the surgeon, also possesses its own merits. For patients with relatively straightforward conditions and clear anatomical structures, U-VATS surgery can swiftly and effectively complete surgical procedures (15, 16). However, the results of this study only showed an increase in the number without any tumor consequences. Previous studies by Nachira D et al. (17) have also shown that higher lymph node production to some extent reflects more thorough lymph node dissection by robot assisted surgery, which can obtain a larger number of lymph nodes and help to more accurately stage tumors. However, this does not directly reflect further related principles that still need further research.

From the perspective of surgical indicators, there were no statistically significant differences between the two surgical approaches in terms of surgical time, intraoperative blood loss, chest tube drainage volume, duration of drainage tube placement, and length of postoperative hospital stay. This indicates that in terms of immediate efficacy, the two surgical methods show similar performance in direct surgical metrics. However, this does not imply that the two approaches are entirely identical in all aspects. In practical surgical settings, robot-assisted surgery may require more time during the preparation phase due to the need for complex equipment calibration and operational procedures. But in recent years, as the Da Vinci robot has moved from the traditional 8,857 punching layout to the present single-hole layout. The installation time is shortened, and the operation time is shortened accordingly (18). On the other hand, while U-VATS surgery is relatively straightforward in operation, it demands a high level of surgical experience from the surgeon and a cooperative surgical team. However, due to the extended learning curve and the need for extensive practices, the mastery of uniportal techniques may take longer (19). Additionally, limitations in instrument flexibility and unclear intraoperative visibility with thoracoscopic instruments hinder further advancements in thoracoscopic technology. Regarding efficacy, although there was no statistically significant difference in efficacy between the two groups, data showed a slightly higher R0 in the da Vinci robot Group at 86.00% compared to 73.47% in the U-VATS group. This may suggest that robot-assisted surgery holds a certain advantage in the thoroughness of tumor resection. The high-definition three-dimensional visualization and precise operational capabilities of the robot system enable surgeons to have a clearer view of the surgical site and more accurately excise tumor tissues, potentially increasing the rate of complete tumor removal and reducing the risk of tumor residue, thereby creating better conditions for long-term patient survival (20, 21). In terms of survival rates, all 99 patients in this study had complete follow-up data. The one-year survival rate was 89.80% in the U-VATS group and 96.00% in the da Vinci robot group, with no statistically significant difference. This suggests that in the short term, the impact on patient survival from these two surgical methods is not markedly different. However, further researches and follow-ups are needed to validate long-term survival rates. As follow-up time extends, one of the current concerns is whether the advantage of robot-assisted surgery in lymph node dissection numbers would translate into a significant improvement in survival rates, which remains an area of interest (22).

Currently, the primary issue with the da Vinci robot system is its high surgical costs. While so far only one manufacturer in the United States produces and sells the da Vinci robot system, new foreign and domestic robots are undergoing accelerated development. The entry of new robot manufacturers is expected to challenge the market pricing of existing robot surgical systems, with domestic research and development of medical surgical robot systems already underway in China (23). Although this study compared the short-term efficacy of robot-assisted and U-VATS right upper lobectomy in treating NSCLC, there are still certain limitations. The study sample was limited to the Nanshishan Hospital of Guangxi Zhuang Autonomous Region, which may restrict the representativeness of the results due to the limited sample size and regional homogeneity, making it challenging to generalize the findings to other regions and populations. Moreover, there may be selection bias in this study, which may affect the accuracy of the results. Therefore, larger scale, multicenter studies are needed in the future to clarify the advantages and disadvantages of surgical procedures using standardized endpoints and long-term follow-up. Additionally, the study focused only on short-term efficacy, lacking long-term follow-up observations on distant outcomes such as 5-year or 10-year survival rates and quality of life. Furthermore, the potential impact of individual patient differences such as genetic characteristics on surgical efficacy was not fully considered. Subsequent researches should aim to expand the sample size, extend follow-up periods, and enhance relevant factor analyses. Finally, inappropriate efficacy endpoint settings may not accurately reflect the true surgical outcome, leading to bias in evaluating surgical advantages or disadvantages and affecting the reliability of conclusions. Incomplete matching methods, such as not fully considering factors such as the patient's basic condition and severity of the disease, can reduce comparability between different groups and interfere with accurate judgments of surgical outcomes and complications. In addition, the complexity of postoperative complications such as lung air leakage, combined with these factors, limits the ability of research to draw comprehensive, accurate, and universal conclusions. Further exploration is needed in the future.

## 4 Conclusion

In summary, in terms of short-term perioperative outcomes, robot assisted and single port thoracoscopic assisted right upper lobectomy are equivalent in the treatment of non-small cell lung cancer. Although robot assisted surgery has shown certain advantages in the number of lymph node dissections, suggesting that it may have greater potential in terms of tumor resection thoroughness, the current conclusions regarding lymph node detection are still exploratory. At the same time, robot assisted surgery faces challenges such as high surgical costs and long doctor training cycles. Although single port thoracoscopic assisted surgery has minimal trauma and fast recovery, it requires high technical skills from doctors. Given the limitations of existing research, there is an urgent need for larger scale, multicenter studies to adopt standardized oncology endpoints and extend follow-up time, in order to further clarify the advantages and disadvantages of the two surgical procedures and provide better treatment options for non-small cell lung cancer patients.

## Data Availability

The original contributions presented in the study are included in the article/Supplementary Material, further inquiries can be directed to the corresponding author/s.
